# Parental Stress and Scalp Hair Cortisol in Excessively Crying Infants: A Case Control Study

**DOI:** 10.3390/children8080662

**Published:** 2021-07-30

**Authors:** Ineke de Kruijff, Ellen Tromp, Mijke P. Lambregtse-van den Berg, Arine M. Vlieger, Marc A. Benninga, Yolanda B. de Rijke, Erica LT. van den Akker

**Affiliations:** 1 Department of Pediatrics, St Antonius Hospital Nieuwegein, 3435 CM Nieuwegein, The Netherlands; a.vlieger@antoniusziekenhuis.nl; 2 Department of Epidemiology and Statistics, St Antonius Hospital Nieuwegein, 3435 CM Nieuwegein, The Netherlands; e.tromp1@antoniusziekenhuis.nl; 3 Departments of Psychiatry and Child and Adolescent Psychiatry, Erasmus MC, 3000 CA Rotterdam, The Netherlands; mijke.vandenberg@erasmusmc.nl; 4 Department of Pediatric Gastroenterology and Nutrition, Emma Children’s Hospital, Amsterdam University Medical Centers, 1105 AZ Amsterdam, The Netherlands; m.a.benninga@amsterdamumc.nl; 5 Department of Clinical Chemistry, Erasmus MC, University Medical Center Rotterdam, 3000 CA Rotterdam, The Netherlands; y.derijke@erasmusmc.nl; 6 Department of Pediatrics, Pediatric Endocrinology, Erasmus MC, University Medical Center Rotterdam, Sophia Children’s Hospital, 3000 CA Rotterdam, The Netherlands; e.l.t.vandenakker@erasmusmc.nl

**Keywords:** hair cortisol, infant, excessive crying, infant colic, stress, father, mother

## Abstract

Background: Caring for an excessively crying infant (ECI) can be stressful for mothers and fathers and is associated with mental and bonding problems. Hair cortisol offers a unique measure for the biological reaction of the body to stress over time. Methods: In this case-control study, scalp hair cortisol concentrations (HCC) were measured using liquid chromatography-tandem mass spectrometry (LC-MS) in 35 mothers and 23 fathers and their ECIs. The control group consisted of 64 mothers and 63 fathers of non-ECIs of similar age. Parental stress, depression, anxiety and bonding were assessed using validated questionnaires. Results: Mean HCC were significantly lower in mothers and fathers of ECIs (2.3 pg/mg, 95% CI 1.8–2.9 and 1.6 pg/mg, 95% CI 1.3–2.0) than that in control mothers and fathers (3.2 pg/mg, 95% CI 3.0–3.7 and 2.9 pg/mg, 95% CI 2.5–3.5). In the total group of parents and within the parents of ECIs, HCC were not associated with negative feelings. In the control group, HCC showed a positive association with stress and depression (r = 0.207, *p* = 0.020 and r = 0.221, *p* = 0.013). In infants, no differences were found in mean HCC between the ECI group and the control group. No associations were found between maternal and infant HCC, paternal and infant HCC and maternal and paternal HCC. Conclusion: Parents of ECIs showed significantly lower HCC than control parents, reflecting a diminished response of the hypothalamic-pituitary-adrenal (HPA) axis. More research is needed to examine whether this decrease in response is pre-existing or caused by excessive infant crying.

## 1. Introduction

Excessive infant crying, often referred to as infant colic, is one of the most distressing challenges for new parents. Recurrent and prolonged periods of infant crying, fussing or irritability, as reported by parents, in otherwise healthy infants under five months of age account for 10–20% of pediatric visits during the first months of life [1,2].

### 1.1. Parental Distress

In these families, a vicious circle is frequently observed in which crying leads to parental stress, which negatively affects the parent–infant relationship and often leads to more crying [3]. For decades, research has focused on maternal feelings and the traditional view of mother as principal caretaker, which is remarkable since in the past decade fathers are more involved than ever in early childcare and there are good reasons to assume that paternal involvement could exert significant influence on both the developing child and the mother. Fortunately, in the last few years, studies have increasingly addressed paternal feelings and have shown that in addition to stress, excessive infant crying has been associated with mental problems such as depression and anxiety and with bonding problems in both mothers and fathers [4,5,6,7,8].

### 1.2. HPA Axis

The neuroendocrine hypothalamic-pituitary-adrenal (HPA) axis and its main downstream effector, the glucocorticoid cortisol, is considered to be a key mediator of the link between chronic stress and mental health problems [9,10]. Limited research supports the association between salivary cortisol levels and excessive infant crying [11]. In one study, the psychological wellbeing of mothers of 24 excessively crying infants (ECIs) was predicted by the infant’s morning salivary cortisol levels and crying intensity [12]. Another study, including 20 ECIs and 20 controls, showed a blunted circadian rhythm of cortisol in the ECI group [13]. This may be an important finding because, in general, flatter diurnal cortisol rhythms across the day are associated with poorer health [14]. However, salivary and blood cortisol levels represent short-term stress responses and momentary stress [15] whereas long-term cortisol measurements are needed to study the chronic stress experienced by parents coping with an ECI.

### 1.3. Scalp Hair Cortisol

Scalp hair cortisol concentrations (HCC) as a marker of long-term cortisol levels have been increasingly studied in the last decade [16,17]. Increased HCC were associated with chronic stress exposure, especially when the exposure was ongoing at the time of the study [9,18]. In contrast, decreased HCC have been found in anxiety disorders, such as post-traumatic stress disorder [9,19]. A broad range of confounders of HCC has been determined in the past decade [9,19].

In children, the study of HCC in relation to stress, trauma exposure and other psychosocial factors [20] showed very heterogeneous results. This may point to difficulties in defining and measuring “stress” and the fact that the HPA axis may be more sensitive to stimuli during specific developmental periods. Persistent stress, such as maternal distress, has been shown related to elevated HCC in young children [21]. Research on HCC has focused on mother-child dyads, studies in the infant group including fathers are, however, lacking. Additionally, it is interesting to investigate more objective methods to measure the severity of the stress experienced by parents and ECIs [22].

We hypothesized that chronic stress experienced by parents and their ECI leads to increased HPA-axis activity signified by increased HCC. In the present study, our aim was to answer the following topics and questions:

- Parental HCC: Do HCC in mothers and fathers with an ECI differ from parents without an ECI (control group)?
- Parental feelings: Is parental HCC associated with experienced stress, depression, anxiety and bonding problems?
- Infant HCC: Do HCC in ECIs differ from that in control infants?
- Association between parental and infant HCC: Is there an association between parental and infant HCC and paternal and maternal HCC?

## 2. Materials and Methods

### 2.1. Study Design and Setting

In this cross-sectional, case-control study, data from two groups of parents were analyzed: mothers and fathers with an ECI and control parents with a non-ECI. Infants aged up to 5 months, who presented with infant colic [1] and their parents were recruited together with their father and mother at the outpatient clinic of the Department of Pediatrics of the St. Antonius hospital in the Netherlands. For the control group, age matched infants were enrolled at infant welfare centers in the neighborhood (75%) and 25% of the controls enrolled at the outpatient clinic of the Department of Pediatrics of the St. Antonius hospital, when they received a routine ultrasound of the hip after breech delivery. Recruitment of the participants occurred between August 2015 and March 2017. Subjects were excluded for the following reasons: parents’ insufficient knowledge of the Dutch language and birth at a gestational age < 36 weeks. Excessively crying infants in the control group (an affirmative answer to the question “Does your child cry excessively?”) were excluded (*n* = 6).

As the participants were not subject to procedures and were not required to follow rules of behavior, the local medical ethical commission deemed that this study was not subject to the Dutch Medical Research Involving Human Subjects (Act WMO). Written informed consent was obtained from all participants.

### 2.2. Measures

#### 2.2.1. Hair Cortisol

At inclusion, hair locks from the posterior vertex were collected from mothers, fathers and infants, by cutting with small surgical scissors as close to the scalp as possible. The hair locks were taped to a paper form with the scalp end marked and then stored in envelopes at room temperature. The proximal 3 cm of the hair was used for a single analysis. In infants whose hair had not grown to 3 cm, at least 1 cm of the hair closest to the scalp was analyzed. Subsequently, hair samples were transferred to glass tubes, weighed (mg), washed by gently shaking them in liquid chromatography-tandem mass spectrometry (LC-MS) grade isopropanol at room temperature and left to dry for at least 48 h. Cortisol was extracted in 1.5 mL LC-MS grade methanol for 18 h at 25 °C in the presence of deuterated cortisol-d3 and it was subsequently cleaned using solid phase extraction. The LC-MS/MS method has been described in detail previously [23,24]. Cortisol concentrations were reported in picograms per micrograms of hair. The lower limit of quantification (LLoQ) of cortisol was <1.0 pg/mg. Measurements below LLoQ were set on 1.0 pg/mg.

#### 2.2.2. Parental Stress, Depression and Anxiety and Parent-Infant Bonding

Experienced parental distress was assessed using validated questionnaires as described in our previous publication [8]. We assessed stress using a Dutch translation of the Perceived Stress Scale (PSS) [25] and depressive symptoms using the Dutch version of the Edinburgh Postnatal Depression Scale (EPDS) [26,27]. Although this instrument was initially developed to investigate mood disturbances in women, the EPDS has proven to be a reliable and valid instrument in screening for depressive symptoms in fathers as well [28,29]. Higher scores indicate higher levels of depressive symptoms.

Symptoms of anxiety were assessed using the short form of the Spielberger State-Trait Anxiety Inventory (STAI), Refs. [30,31,32] and problems in the parent-infant relationship were assessed using the Dutch version of the Postpartum Bonding Questionnaire (PBQ) [33,34,35]. Originally the PBQ has been developed and validated for measuring mother-infant bonding. Since a questionnaire about father-infant bonding has not yet been developed, the PBQ was also used for fathers in this study. Higher sum scores in the used questionnaires indicate increased levels of parental distress and problems with bonding.

#### 2.2.3. Infant Crying Behavior

Parents were asked to record the duration of infant crying in a diary for three consecutive days [36]. For this study, crying episodes lasting for at least 10 min were recorded. Because crying intensity may reflect greater physiological or psychological stress than duration of crying, we additionally asked the parents to record the infant crying volume and intensity on scales ranging from 1 to 9, representing, respectively, barely audible to very loud crying and no facial expression to painful grimacing while crying [11].

#### 2.2.4. Confounders and Mediators

The following factors were obtained using standardized questions and included as potential confounders: information about demographic and hair characteristics, smoking, medication use and course, complications and experience of pregnancy and delivery. The pregnancy and delivery experience was queried using the question “How did you experience the pregnancy/delivery?” The answers were reported on a scale ranging from 1 to 5, representing very positive to very negative. Furthermore, pre-existing factors contributing to the parents’ current emotional state, such as recent stressful events, emotional problems during pregnancy and current psychiatric treatment, were assessed using standardized questions.

### 2.3. Statistical Analysis

Data are presented as means (±standard deviation (SD), medians (range or interquartile range), or counts (%), where appropriate. Sum scores of the four questionnaires were obtained and an average was calculated for both mothers and fathers. A maximum of 33.3% missing data within one subject was accepted for these questionnaires and corrected for by taking the participants’ mean of the completed questions.

Sociodemographic and hair characteristics were compared between the ECI and control groups with the Chi-square test/Fisher’s exact test and independent samples *t*-test, or the Mann–Whitney U test in case of non-parametric data. As HCC have a skewed distribution, log-transformation was applied to achieve a normal distribution. In presenting the results, the log HCC were recalculated in the real HCC and the corresponding 95% confidence intervals (CI).

Mean HCC in mothers, fathers and infants were compared between the case and control group using the independent samples *t*-test (unadjusted mean and 95% CI) and linear regression analysis. In these analyses, adjustments for parents (age, high education level, ethnicity and emotional/psychiatric problems during pregnancy) and adjustments for infant factors (age, male sex, medication use of infant and psychotropic medication use of mother) were applied. Potential confounders were identified by a 10% change-in-estimate criterion for estimated associations or determination as confounders in previous studies.

Multivariate linear regression analyses were also used to investigate the association between the HCC in mothers, fathers and both parents to parental stress, depression, anxiety and bonding scores with and without adjustments for potential confounding factors and with and without splitting for case and control groups.

Additionally, we used multivariate linear regression analyses to investigate the association between the HCC in mothers, fathers and both parents to infant crying duration, volume and intensity with and without adjustments for potential confounding factors and with and without splitting for case and control groups.

The association between maternal and infant HCC, paternal and infant HCC and maternal and paternal HCC in both the case and control samples was investigated using linear regression analysis, with and without adjustments for potential confounding factors and with splitting for case and control groups. A *p*-value of <0.05 was considered statistically significant. All analyses were performed using IBM SPSS (IBM SPSS Statistics for Windows, Version 26.0. Armonk, NY: IBM Corp. 

## 3. Results

**Figure 1:**
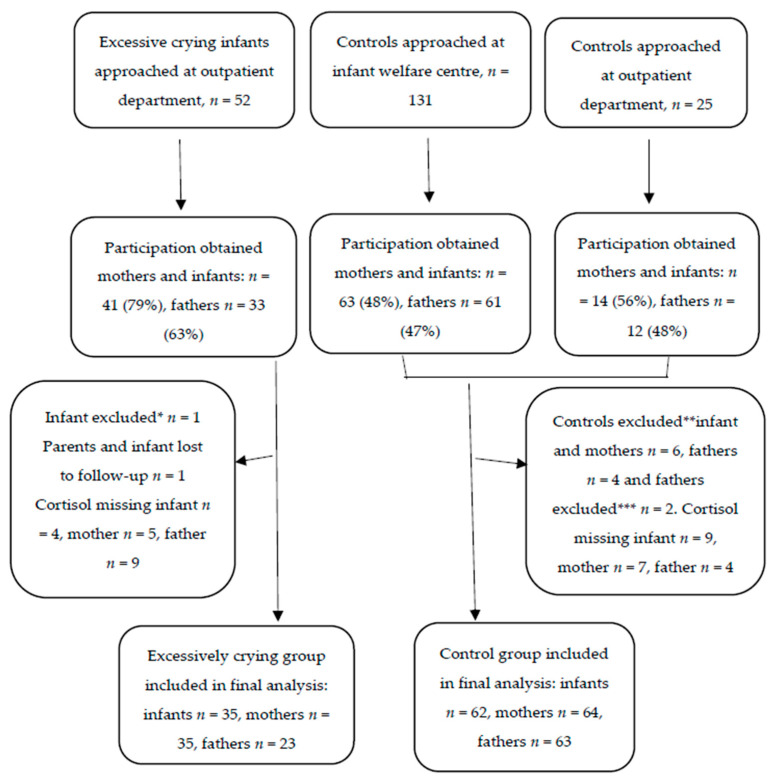
Flow diagram of inclusions. * Case infant excluded because of gestational age <36 weeks, ** Controls excluded because of one of the parents experienced their infant as excessively crying based on the questionnaire, *** Control parents excluded because of oral corticosteroid use.

A total of 208 families (both cases and controls) were approached for participation, of whom 99 mothers, 86 fathers and 97 infants, were included in the final hair cortisol analysis (Figure 1).

The sociodemographic characteristics of the participating families are shown in Table 1.

There was no difference in hair color and delivery mode between groups. In both parents, hair bleaching and dyeing occurred more often in the excessive crying group (43% vs. 20%, *p* = 0.017), but it was not identified as a confounder.

Table 2 shows parental distress: the reported levels of stress, depression, state anxiety and bonding behavior of both parents and crying behavior of their infant in the ECI group and control group.

The mothers and fathers with ECIs reported significantly more stress, depression, anxiety, bonding problems, crying time and intensity than control mothers and fathers.

Parental HCC and infant HCC in the case group of ECIs and that in controls are shown in Table 3.

### 3.1. Parental HCC

Mean HCC in mothers were significantly lower in the ECI group (2.3 pg/mg, 95% CI 1.8–2.9) than in the control group (3.2 pg/mg, 95% CI 3.0–3.7). Mean HCC in fathers was also significantly lower in the ECI group (1.6 pg/mg, 95% CI 1.3–2.0) than in the control group (2.9 pg/mg, 95% CI 2.5–3.5). Adjustment for confounders did not change these results.

### 3.2. Parental Feelings

Multivariate linear regression analyses showed no significant association between HCC in mothers, fathers or both parents and stress, depression, anxiety and bonding sum scores. The group of parents with an ECI showed no significant associations between HCC and the sum scores of stress, depression, anxiety and bonding. However, HCC in the control group parents showed a statistically significant increase (r = 0.207, *p* = 0.020) with increasing PSS sum scores. A similar positive association was found between parental HCC and EPDS sum scores in the control group of control parents (r = 0.221, *p* = 0.013). No association was found between HCC and STAI or PBQ scores. Adjustment for confounders did not change these results.

### 3.3. Infant HCC

No differences in HCC were found between ECIs and control infants (32 pg/mg, 95% CI 25–41 and 34 pg/mg, 95% CI 26–44).

### 3.4. Association between Parental and Infant HCC

The maternal HCC and infant HCC showed a non-significant association in both the case- and control groups (r = 0.25 versus r = 0.18). The correlation between paternal HCC and infant HCC in the case- and control groups (r = 0.16 versus r = −0.18) and that between maternal HCC and paternal HCC in the case- and control groups (r = −0.16 versus r = 0.05) were also not significant. Multivariate linear regression analyses showed no significant association between HCC in mothers, fathers or both parents and infant crying duration, volume and intensity.

## 4. Discussion

In our study, our first aim was to study parental HCC and we showed that parents with an ECI had lower HCC compared with control parents, while the HCC in control parents were in the normal range, as found in healthy adults [37,38,39]. Based on previous hair cortisol studies reporting increased HCC after stress exposure, we expected higher, instead of lower mean HCC in parents of ECIs, especially because the excessive crying continued at the time of the study [18,40]. One explanation for this seemingly contradictory result could be that the experience of infant crying is a different form of stress than somatic stress. Flattening of the cortisol diurnal slope or dampening of the cortisol response is a well-known phenomenon in psychological studies [9,41] and has, for example, been associated with psychosocial stress in women with preexistent psychopathology [42]. Studies of HCC after traumatic events have shown both negative and positive associations between HCC and trauma, indicating that the strength and direction of the association are moderated not only by type and timing of trauma but also by study characteristics, like racial distribution, clinical diagnosis/or non-clinical diagnosis and features of the publication (for example the geographical region of the study) [43]. The lowering of the parental HCC could not be explained by certain characteristics of the infant crying; the duration, volume and intensity of crying showed no correlation with parental HCC in our study.

In order to identify whether the type of psychological stress influenced parental HCC, our second aim was to study the association between HCC and parental feelings of stress, depression, anxiety and bonding problems. Parents of an ECI significantly experienced more stress, depression, anxiety and bonding problems than the control parents. These stressful feelings may have resulted in the downregulation of the HPA system. Still, since we did not find an association between the severity of these feelings and HCC in ECI parents, we speculate that the downregulated parental HPA system could already have been present during pregnancy. It is known that maternal anxiety during pregnancy is related to an excessive crying infant [6]. This apparent downregulation of the parental HPA system in ECI parents may result from early adverse life events inducing persistent changes in the HPA axis, which may predispose these parents to the development of mood and anxiety disorders in this period of their lives [44].

Control parents with increasing stress and depression scores had higher HCC. In contrast, in parents of ECIs, no association was found between HCC and experienced stress, depression, anxiety and bonding problems. These findings also suggest a dampening of responsivity of the HPA axis to stress and depression in these parents.

To the best of our knowledge, this is the first study evaluating HCC in fathers of both healthy and excessive crying infants. In accordance with findings in mothers of excessive crying infants, we found the same significant lowered HCC in the exposed group and no association with infant HCC. We found no association with maternal HCC. In contrast, one cross-sectional study reported an association of HCC in 6-year-old children and their mother and father [45]. Future research should investigate if the same mechanism as in mothers, where anxiety during pregnancy is related to excessive crying [6], plays a role in fathers of infants with excessive crying and which other factors could contribute to the lowered HCC.

Our third aim was to investigate differences in HCC in ECIs and control infants. In contrast to the findings in their parents, no differences in infant HCC were found between the two groups. One potential explanation is that excessive crying is not a stressor to the infant itself. This is in line with a study looking into the correlation between neonatal hair cortisol levels and infant distress [46]. Another explanation could be that the age range of the infants plays a role. HCC levels in infants are very high at birth and decrease in the first months of life. Our recent study on normal HCC values in children [24] showed that HCC in infants are more than 10-fold higher than that in adults, have wide reference ranges and show a sharp decline in the first three months of life. Therefore, future studies on HCC in infants need to use a smaller age range (per month) and larger sample sizes per age to identify subtle concentration changes. Furthermore, there may be other factors moderating the association between stressors and physiological stress in infants, such as parental care and caregiver relationships [47], which need to be investigated in larger population cohorts.

Our last aim was to study the association between parental and infant HCC. No correlation was found between maternal and infant HCC, paternal and infant HCC and maternal and paternal HCC within both groups, in contrast to earlier studies [48,49]. However, our results are in line with a more recent study, in which mother-infant dyads subjected to severe psychiatric disorders also showed no correlation between maternal and infant HCC [50].

### 4.1. Strengths and Limitations

The major strength of our study is the unique sample of parents and infants experiencing the stressor “excessive crying”, in a controlled design, including the measurement and adjustment of a broad range of confounders. Existing literature concerning excessive infant crying has focused primarily on mothers’ perceptions, while feelings and caring for an excessively crying infant can be challenging for both parents. Therefore, the inclusion of fathers is another strength of this study.

This study also has several limitations. First of all, our study is limited by the cross-sectional design, which prevents us from establishing causality. Secondly, the age range of the infants might have been too broad to identify a difference between HCC in ECIs and control infants. The mean infant age differed slightly between groups, for which we corrected in the analyses. Thirdly, in the control group, selection bias cannot be excluded as only half of the parents approached were willing to participate. Fourthly, the PBQ questionnaire was not designed for measuring father–infant bonding and the time frame of hair cortisol and questionnaires did not fully overlap. We investigated the association between mean HCC in 3 cm of hair—representing 3 months—while parental distress was examined at the time of the questionnaire (STAI, PBQ), during the past week (EPDS) and in the last month (PSS). Prospective studies with larger samples, smaller age ranges of ECIs, stress questionnaires during pregnancy and the first months after birth and addressing additional factors such as parental care and caregiver relationships are needed to examine whether HCC can serve as a stress marker experienced by parents caring for ECIs. Future research projects on establishing evidence-based management strategies for excessive crying/infant colic may benefit from this marker.

### 4.2. Conclusions

In conclusion, this study shows that HCC in the parents of ECIs is significantly lower than that in control parents. In addition to the specific stressor of excessive infant crying, the characteristics of the parental sample, experiencing more stress, anxiety, depression and bonding problems, potentially contributed to the difference we observed. We speculate that the downregulation of the parental HPA axis already started during pregnancy, related to prenatal anxiety and may even represent the cause of excessive crying. We conclude that integrated care for both fathers and mothers should ideally start during early pregnancy to reduce feelings of stress and anxiety, which could positively contribute to preventing excessive crying in their infants.

## Figures and Tables

**Table 1:** Sociodemographic characteristics.

	Excessive Crying Mean (SD) or % and (N)	Control Mean (SD) or % and (N)	*p*-Value
Infants’ age (w)	8.54 (3.37) (35)	10.19 (3.95) (62)	0.040
Male gender	57.1 (20/35)	45.2 (28/62)	
Gestational age at birth (w)	38.9 (1.3) (35)	39.3 (1.4) (62)	0.257
Birthweight (g)	3312 (534) (35)	3433 (555) (62)	0.150
Feeding status			0.300
- Exclusive breastfeeding	20.0 (7/35)	46.8 (29/62)	
- Formula only	80.0 (28/35)	40.3 (25/62)	
- Breast milk and formula	0	12.9 (8/62)	<0.001
Use of medication infant	31.4 (11/35)	0 (0/62)	<0.001
- Acid reflux treatment			
Use of medication mother - Psychotropic medication	14.13 (5/35)	3.1 (2/64)	0.093
Age (y)			
- Mother	31.2 (3.2) (35)	31.9 (4.6)(64)	0.354
- Father	33.4 (4.6) (23)	34.5 (4.9)(63)	0.344
Ethnicity (Dutch-Caucasian)			
- Mother	100 (35/35)	90.6 (58/64)	0.087
- Father	87 (20/23)	93.7 (59/63)	0.378
Educational level of mother			
- Secondary school or less	5.7 (2/35)	4.7 (3/64)	
- Secondary vocational education	48.6 (17/35)	17.2 (11/64)	0.004
- Higher professional education	28.6 (10/35)	43.8 (28/64)	
- University	17.1 (6/35)	34.4 (22/64)	
Educational level of father			
- Secondary school or less	21.7 (5/23)	9.5 (6/63)	
- Secondary vocational education	30.4 (7/23)	33.3 (21/63)	0.298
- Higher professional education	26.1 (6/23)	30.2 (19/63)	
- University	21.7 (5/23)	27.0 (17/63)	
Current Smoking			
- Mother	14.7 (5/34)	4.8 (3/63)	0.124
- Father	17.4 (4/23)	15.9 (10/63)	1.000
Emotional/psychiatric problems pregnancy			
- Mother	25.7 (9/35)	10.9 (7/64)	0.056
- Father	8.7 (2/23)	1.6 (1/63)	0.173
Current psychiatric treatment			
- Mother	14.3 (5/35 ^1^)	3.1(2/64)	0.093
- Father	8.7 (2/23)	1.6 (1/63)	0.173
Experienced stressful events			
- Mother	54.3 (19/35)	38.1 (24/63)	0.122
- Father	52.2 (12/23)	41.0 (26/63)	0.367
Negative experience of pregnancy			
- Mother	14.3 (5/35)	6.5 (4/62)	0.277
- Father	8.7 (2/23)	1.6 (1/61)	0.181
Negative experience of delivery			
- Mother	14.7 (5/34)	14.5 (9/62)	1.000
- Father	17.4 (4/23)	3.3 (2/61)	0.045

^1^ two mothers > diagnoses.

**Table 2:** Reported levels of stress, depression, state anxiety and bonding behaviour of mother and father and crying behaviour of infant in the excessive crying and control group.

Variable	Excessive Crying Mean (SD) and (N)	Control Mean(SD) and (N)	*p*-Value
Stress (PSS)			
- *Mother*	25.2 (8.1) (33)	14.1 (6.9) (64)	<0.001
- *Father*	21.0 (6.3) (20)	16.2 (6.1) (62)	0.003
Depression (EPDS)			
- *Mother*	8.8 (5.2) (34)	3.8 (3.2) (64)	<0.001
- *Father*	5.4 (4.3) (20)	2.8 (2.9) (62)	0.016
State Anxiety (STAI)			
- *Mother*	45.9 (12.3) (34)	31.6 (9.3) (64)	<0.001
- *Father*	42.1 (10.0) (21)	31.6 (7.5) (62)	<0.001
Bonding behaviour (PBQ)			
- *Mother*	15.2 (8.2) (16)	4.8 (4.4) (64)	<0.001
- *Father*	18.0 (8.4) (10)	7.9 (5.9) (62)	<0.001
Crying duration (min)	112 (78) (31)		
- during the day	74 (53) (31)	26 (24) (61)	<0.001
- during the night		14 (15) (61)	<0.001
Intensity of crying	5.9 (1.9) (13)	2.6 (1.6) (61)	<0.001
Volume of crying	6.0 (1.8) (13)	3.5 (5.3) (61)	0.096

**Table 3:** Hair cortisol concentration mean (±95% CI) in cases versus controls infant, mother and father.

Characteristics	Unadjusted Mean (95% CI)	Log Beta	*p*-Value	Log Adjusted Beta	*p*-Value	Log Adjusted Beta	*p*-Value
HCC infant (pg/mg)							
Control (*n* = 62)	34.0 (26.3–44.0)	Ref		Ref *			
Exc crying (*n* = 35)	32.1 (25.1–40.9)	−0.06	0.762	−0.14	0.510		
HCC mother (pg/mg)							
Control (*n* = 64)	3.2 (3.0–3.7)	Ref		Ref **		Ref **	
Exc crying(*n* = 35)	2.3 (1.8–2.9)	−0.35	0.009	−0.41	0.002	−0.40	0.003
HCC father (pg/mg)							
Control (*n* = 63)	2.9 (2.5–3.5)	Ref		Ref **		Ref ***	
Exc crying (*n* = 23)	1.6 (1.3—2.0)	−0.60	<0.001	−0.62	<0.001	−0.60	0.001

* corrected for infant factors (age, male sex, medication use, psychotropic medication use of mother); ** corrected for demographic factors (age, high education level, ethnicity); *** corrected for emotional/psychiatric problems during pregnancy.

## Data Availability

The data presented in this study are available on request from the corresponding author. The data are not publicly available due to privacy and ethical reasons.

## Abbreviations

CI; confidence intervals, HCC; hair cortisol concentrations, HPA; hypothalamic-pituitary-adrenal, EPDS; Edinburgh Postnatal Depression Scale, LC-MS; liquid chromatography-tandem mass spectrometry, LLoQ; lower limit of quantification, PBQ; Postpartum Bonding Questionnaire, PSS; Perceived Stress Scale, STAI; Spielberger State-Trait Anxiety Inventory; SD standard deviation.
